# Juvenile diet quality and intensity of sexual conflict in the mite *Sancassania berlesei*

**DOI:** 10.1186/s12862-020-1599-5

**Published:** 2020-03-12

**Authors:** Aleksandra Łukasiewicz

**Affiliations:** Evolutionary Biology Group, Faculty of Biology, Adam Mickiewicz University Poznań, ul. Uniwersytetu Poznańskiego 6, 61-614 Poznań, Poland

**Keywords:** Sexual conflict, Behavioural ecology, Diet quality, Condition dependence, Male-male competition, Male-induced harm, *Sancassania berlesei*

## Abstract

**Background:**

Differing evolutionary interests of males and females may result in sexual conflict, whereby traits or behaviours that are beneficial for male reproductive success (e.g., traits related to male-male competition) are costly for females. Since sexual conflict may play an important role in areas such as speciation, population persistence or evolution of life history traits, understanding what factors modulate the intensity of sexual conflict is important. This study aims to examine juvenile diet quality as one of the underestimated ecological factors that may affect the intensity of sexual conflict via individual conditions. I used food manipulation during the development of the mite *Sancassania berlesei* to investigate the effects on male reproductive behaviour and competitiveness, male-induced harm to female fitness and female resistance to this harm.

**Results:**

Males that were exposed to low-quality food started mating later than the control males, and number of their mating attempts were lower compared to those of control males. Moreover, males from the low-quality diet treatment sired fewer offspring under competition than males from the control treatment. However, the fitness of females exposed to males reared on a poor diet did not differ from that of females mated with control males. Furthermore, female diet quality did not alter their resistance to male-induced harm.

**Conclusion:**

Overall, diet quality manipulation affected male reproductive behaviour and mating success. However, I found no evidence that the intensity of sexual conflict in *S. berlesei* depends on male or female conditions. Investigating a broader range of environmental factors will provide a better understanding of sexual conflict dynamics and its feedback into associated evolutionary mechanisms.

## Background

For decades, sexual reproduction has been reputed as an act of cooperation and harmony between the two sexes. However, a growing body of evidence shows that the evolutionary interests of males and females not always overlap, which may lead to sexual conflict [1–4]. This sexual conflict may lead to sexually antagonistic evolution of traits and/or behaviours that increase the fitness of one sex, usually males, at the expense of the fitness of the other sex, usually females, who are selected to overcome these male adaptations [1]. When sexual conflict is associated with traits determined by different genes in the two sexes, sexually antagonistic selection may favour traits, such as adaptations to sexual competition, that increase male reproductive success even if they have negative effects on their partners’ fitness [2]. To increase their own fitness, males can evolve traits allowing them to induce harm during copulation [5], for example, by traumatic insemination [6] or transfer of toxic seminal fluids along with sperm [7, 8]. In response to these adaptations, sexually antagonistic coevolution may drive the evolution of traits that increase female fitness through enhanced resistance to harassment or coercion [9, 10]. Such intersexual conflict may therefore result in an evolutionary ‘arms race’ between males and females in which any benefit obtained by one sex negatively affects the fitness of the other [2, 11, 12].

Since, except in strict monogamy, optimal fitness of males and females cannot be simultaneously achieved [1, 3, 4, 13], sexual conflict may be common across a wide range of sexual species [3, 14, 15] and applies to most mating systems [16]. Studies conducted on a wide range of taxa provided evidence that sexual conflict may contribute to rapid population divergence [17, 18] as well as the evolution of reproductive isolation [14, 19], ultimately leading to speciation [20]. However, sexual conflict may also reduce population productivity [21, 22], possibly increasing the risk of extinction [23]. Understanding the impact of sexual conflict on the aforementioned key evolutionary processes requires an understanding of the factors that affect its intensity.

A number of recent studies have emphasised the need for understanding sexual conflict in its ecological context [24–27]. For example, Gomez-Llano et al. (2018) showed that the interaction of species composition (sympatric or allopatric) and male density in damselflies affects harassment by males and female survival during mating [28]. Sexual conflict may also be modulated by abiotic factors such as the physical structure of the habitat (small and simple or large and complex), which, for example, affects the frequency of sexual interaction and male-induced harm in *Drosophila melanogaster* [29]. It was also shown that, in the same species, temperature variation has a dramatic impact on male-induced harm towards females via behavioural plasticity [30]. The results of all of these studies indicate that research performed in uniform, simple environments may have led to a biased perception of the importance of sexual conflict. To fully appreciate the evolutionary significance of sexual conflict and its effect on natural populations, it is important to conduct studies that look beyond homogeneous and optimal laboratory conditions.

Another factor that may potentially modulate the intensity of sexual conflict is the quality of food resources. Diet availability and quality are the most commonly used factors in eco-evolutionary research due to their well-studied impact on life expectancy [31–37] and reproductive performance [31, 33, 37–39], including sex-specific life history traits [36], reproductive rates [40], sex-specific costs of mating [41], and components of the ejaculate [42], as well as because diet is easy to manipulate under laboratory conditions. Overall, variation in the quality of food resources can affect traits involved in sexual conflict or other traits that may in turn modify the intensity of this process.

Since diet affects each individual’s condition, expectations regarding how food quality may affect the intensity of sexual conflict can be based on a condition dependence model. Condition dependence models imply that high-condition males should invest in costly sexually selected traits if it would be evolutionarily beneficial. Examples of condition dependence of male sexually selected traits are well documented [43–45]. If such traits are involved in sexual conflict, the intensity of the latter might also be condition dependent. A study conducted on *D. melanogaster* seems to support this hypothesis. Friberg and Arnqvist (2003) have shown that the largest males are both the most attractive and the most harmful to females [46]. However, to fully understand how food quality affects the intensity of sexual conflict, comprehensive studies documenting the condition dependence of male competitiveness, along with the effects on female fitness and female condition on resistance to the harmful effects of males, are needed.

This study aims to fill this gap using the mite *Sancassania berlesei,* a species in which intense sperm competition was demonstrated [47] along with negative effects of multiple mating events on female fecundity and lifespan [48]. Since previously observed costs of mating in *S. berlesei* [48] might have been elevated by a strongly male-biased sex ratio, I first confirmed that sexual conflict occurs under unbiased sex ratios. I then examined how the quality of food affects male competitiveness and the intensity of sexual conflict. I used a high-quality and low-quality feeding regime during development to test predictions that (1) good condition of males is associated with increased reproductive competitiveness, which leads to increased male reproductive success; (2) male-induced harm is decreased when females mate with low-condition males; and (3) female resistance to this harm is linked to the female condition.

## Results

### Effect of mating frequency on female fitness

**Fig. 1:**
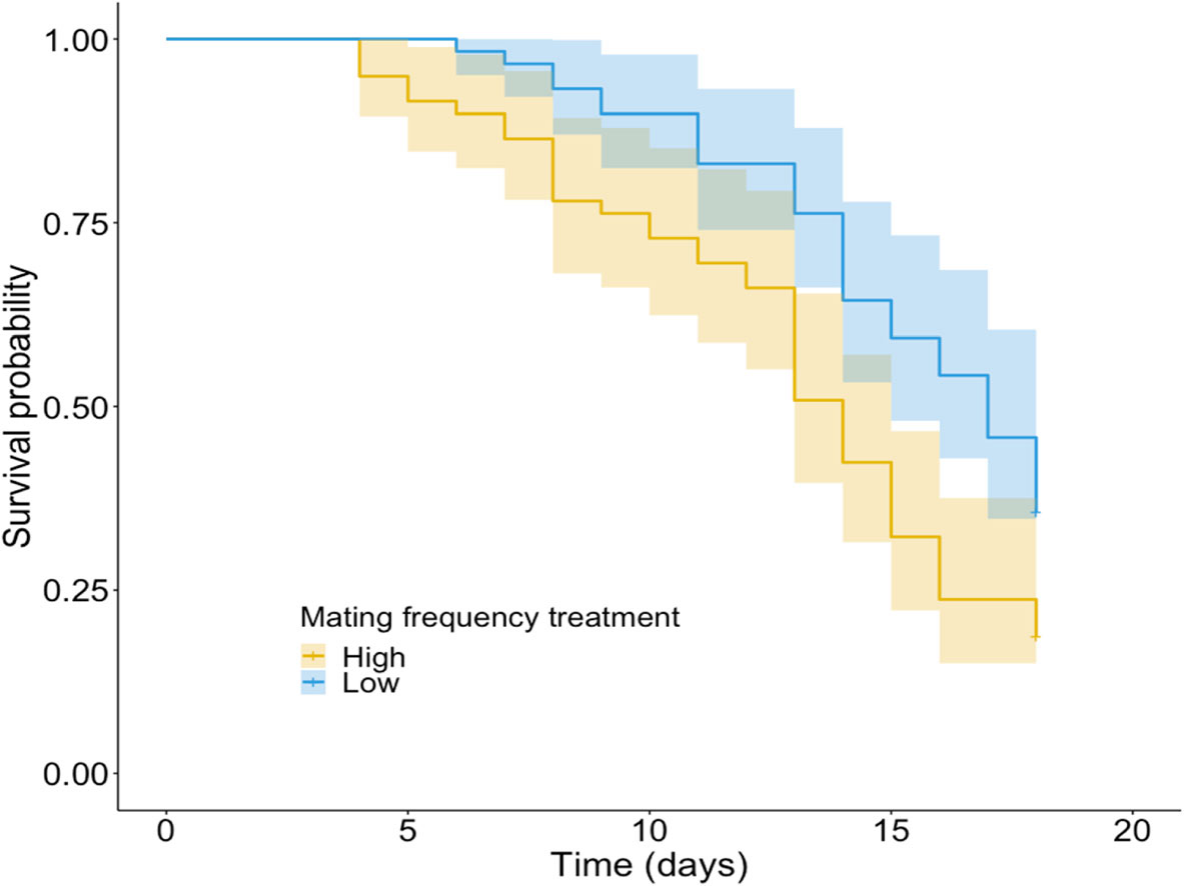
Effect of mating frequency on female lifespan. Kaplan-Meier survival plots for females maintained in high mating frequency and low mating frequency groups

The female lifespan was significantly shorter when the mating frequency was higher (*z* = − 3.78, *p* < 0.001; Table 1, Fig. 1) and interacted with the male morph (male morph * mating frequency interaction for female lifespan: *z* = 2.07, *p* = 0.039; Table 1). Further analysis of this interaction revealed that mating frequency decreased female lifespan but only when females were mated with scrambler (*z* = − 3.74, *p* < 0.001, Fig. S1, Table S1) and not a fighter (*z* = − 0.91, *p* = 0.36, Fig. S1, Table S1) male.

**Table 1:** Results of a mixed-effects Cox regression model of female survival probability as a function of mating frequency, with experimental treatment, male morph, number of mated males and interaction between treatment and male morph as fixed effects and male ID as a random effect

**Fixed effect:**	*coef.*	*s.e.*	*z*	*p*
Treatment	−1.26	0.33	−3.78	**< 0.001**
Male morph	−0.65	0.35	−1.86	0.064
Mating with more than one male	−0.29	0.32	−0.90	0.370
Treatment*Male morph	0.95	0.46	2.07	**0.039**
**Random effect:**	*variance*	*std. dev.*		
Male ID	0.432	0.658		

*Abbreviations*: *coef.* Coefficient, *s.e.* Standard error, *std. dev*. Standard deviation; *p* < 0.05 highlighted in bold

**Fig. 2:**
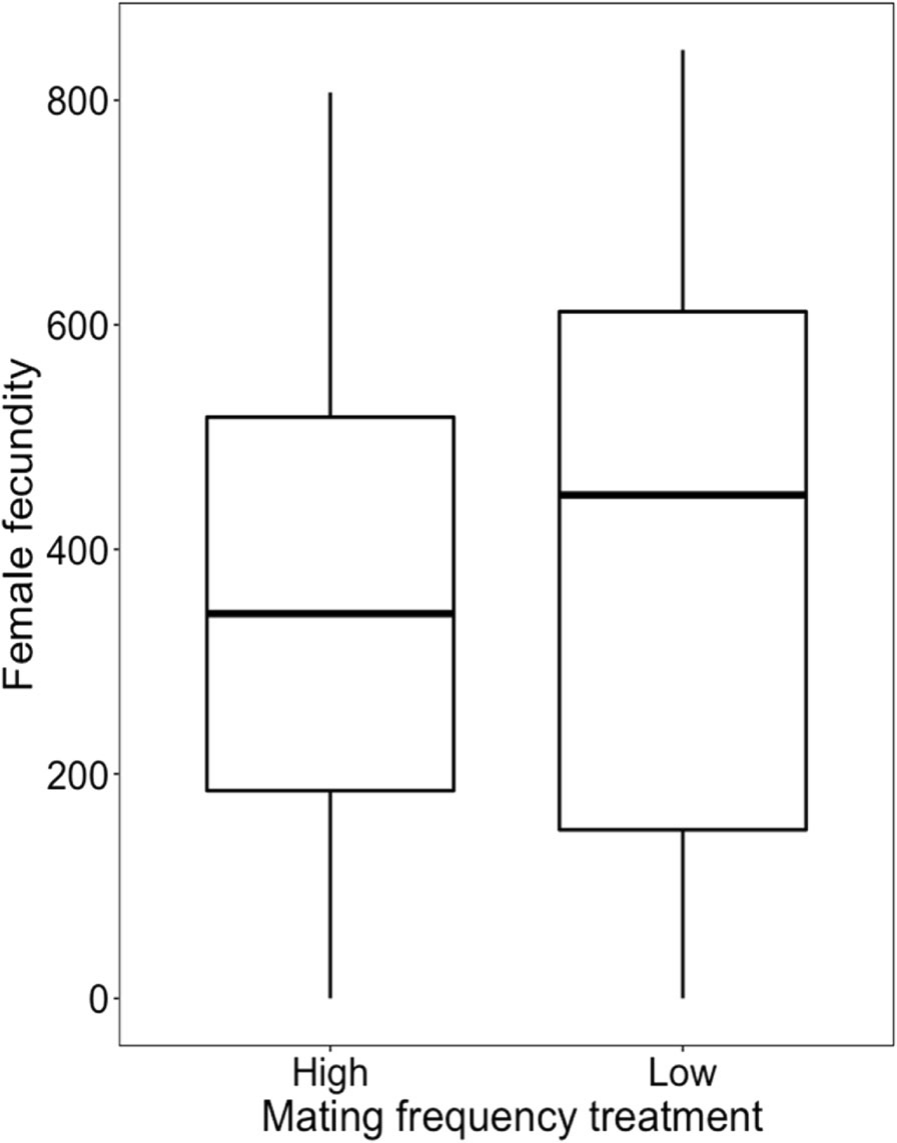
Effects of mating frequency on female reproductive output. Boxplots showing the distribution of the number of eggs laid (between the 6th and 11th days of the experiment) by females in the high mating frequency and low mating frequency treatments. The box encloses values between the first and third quartiles of the data (the inter-quartile range, IQR), while the horizontal bar within the box indicates the median. Whiskers extend from the box to the largest/smallest values that are within 1.5Χ the IQR of the box. Values outside that range are outliers and are indicated by circles

Female lifespan was a significant predictor of fecundity (GLMM: *z* = 5.83, *p* < 0.001, Table 2), but I did not detect any significant effect of either treatment (GLMM: *z* = − 0.69, *p* = 0.489; Table 2, Fig. 2) or male morph (GLMM: *z* = 1.33, *p* = 0.183; Table 2, Fig. 2) on female fecundity while controlling for lifespan.

**Table 2:** Results of a generalised linear mixed model with negative binomial error distribution of female fecundity as a function of mating frequency, with experimental treatment, male morph, number of mated males and female lifespan as fixed effects and male ID as a random effect

**Fixed effect:**	*coef.*	*s.e.*	*z*	*p*
Treatment	−0.153	0.22	−0.69	0.489
Male morph	0.288	0.22	1.33	0.183
Mating with more than one male	−0.435	0.24	−1.80	0.072
**Lifespan**	0.224	0.04	5.83	**< 0.001**
**Random effect:**	*variance*	*std. dev.*		
Male ID	1.125 × 10^−7^	< 0.001		

*Abbreviations*: *coef.* Coefficient, *s.e.* standard error, *std. dev*. Standard deviation; *p* < 0.05 highlighted in bold

### Manipulation of juvenile diet quality

Manipulation of diet quality during development influenced male body size. Body length differed significantly between the treatments (*U* < 0.00*, n*_*1*_ *= 20, n*_*2*_ *= 21*, *p* < 0.001) - males treated with a low-quality diet were smaller than the control males.

### Effect of juvenile diet quality on male sexual behaviour and competitiveness

**Fig. 3:**
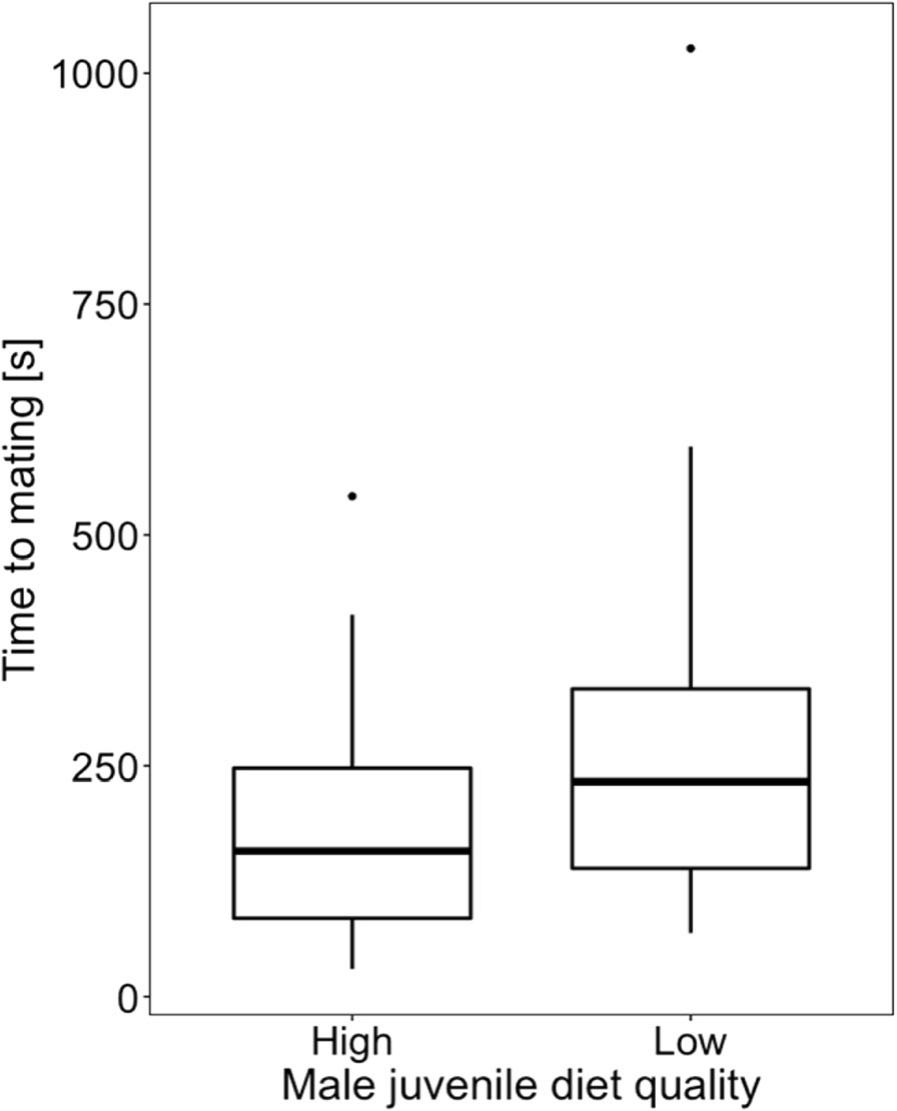
The effect of male diet quality during development on time to start of first copulation. The box encloses values between the first and third quartiles of the data (the inter-quartile range, IQR), while the horizontal bar within the box indicates the median. Whiskers extend from the box to the largest/smallest values that are within 1.5Χ the IQR of the box. Values outside that range are outliers and are indicated by circles

**Fig. 4:**
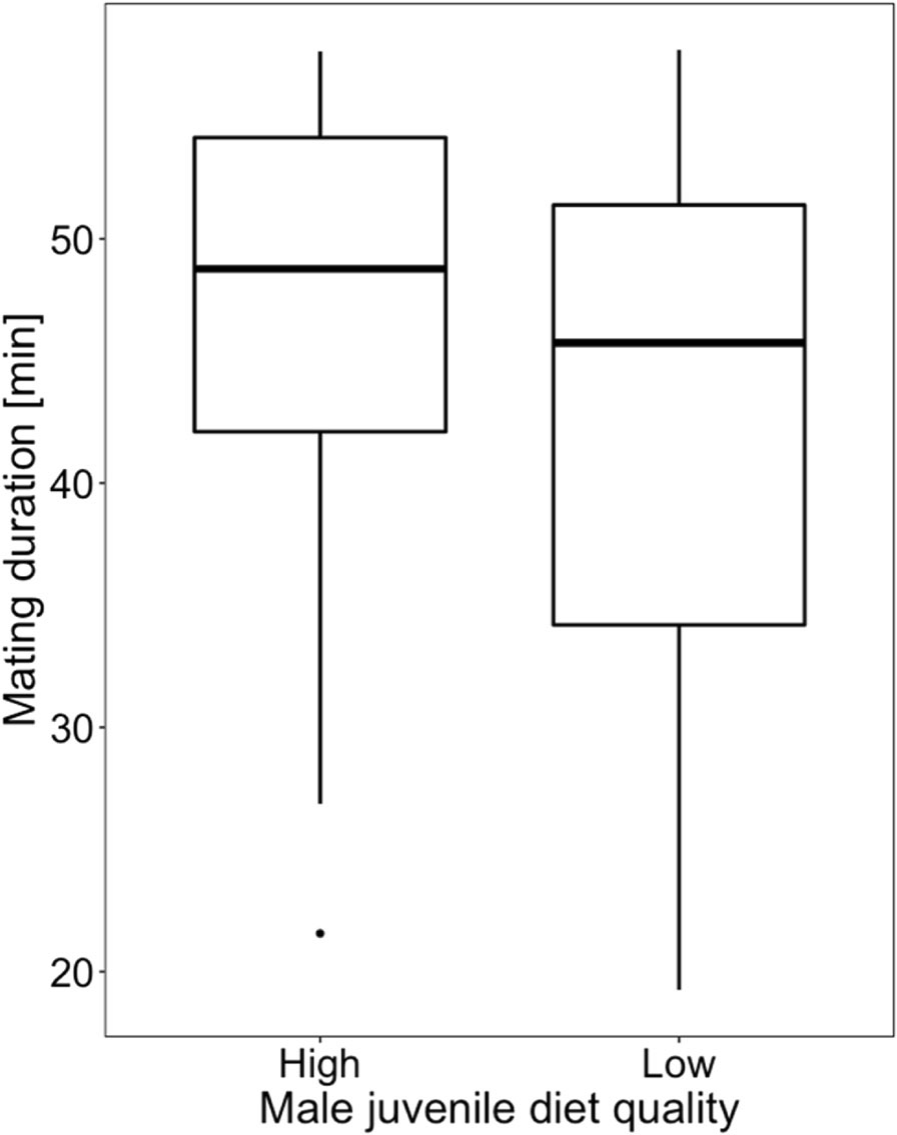
Effect of male diet quality during development on mating duration within 60 min of observation. The box encloses values between the first and third quartiles of the data (the inter-quartile range, IQR), while the horizontal bar within the box indicates the median. Whiskers extend from the box to the largest/smallest values that are within 1.5Χ the IQR of the box. Values outside that range are outliers and are indicated by circles

**Fig. 5:**
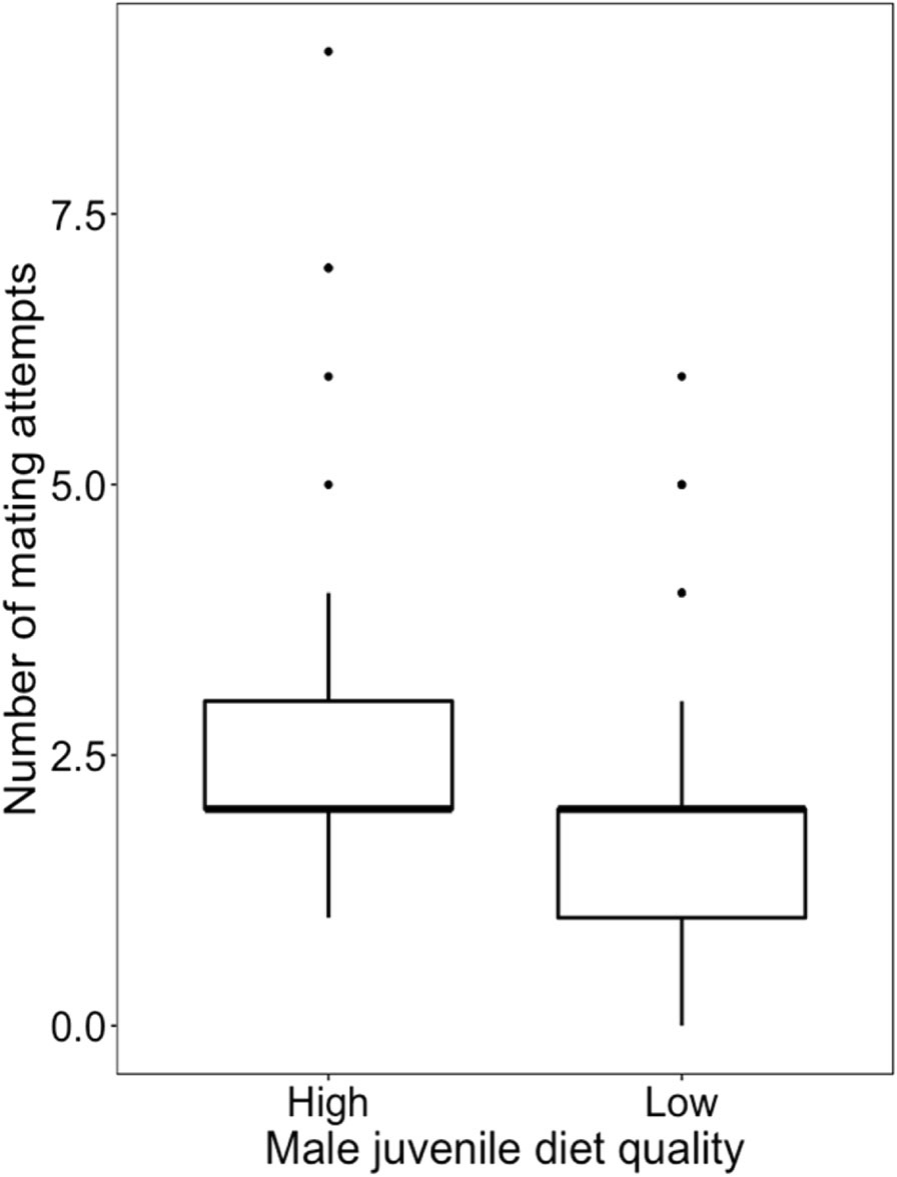
Effect of male diet quality during development on the number of mating attempts within 60 min of observation. The box encloses values between the first and third quartiles of the data (the inter-quartile range, IQR), while the horizontal bar within the box indicates the median. Whiskers extend from the box to the largest/smallest values that are within 1.5Χ the IQR of the box. Values outside that range are outliers and are indicated by circles

**Fig. 6:**
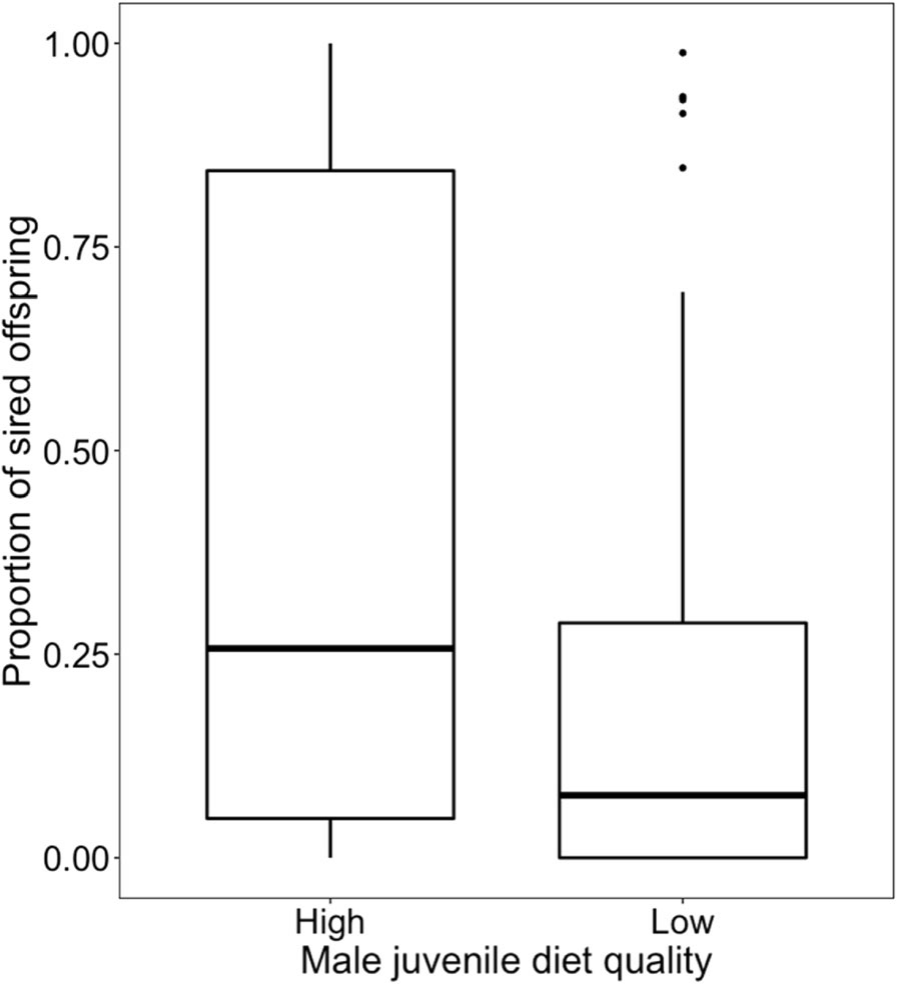
Effect of male diet quality during development on male competitiveness. The box encloses values between the first and third quartiles of the data (the inter-quartile range, IQR), while the horizontal bar within the box indicates the median. Whiskers extend from the box to the largest/smallest values that are within 1.5Χ the IQR of the box. Values outside that range are outliers and are indicated by circles

Males treated with a low-quality diet began mating significantly later than the control males (*t*_*73*_ = 2.899, *p* = 0.005; block *p* = 0.101; Fig. 3). Mating duration did not significantly differ between the treatments (*t*_73_ = − 1.511, *p* = 0.134; block *p* = 0.001; Fig. 4), however the number of copulation attempts was lower in low-quality male group than in control one (*z* = − 2.34, *p* = 0.019; block *p* = 0.055; Fig. 5). Male competitiveness was lower in the low-quality treatment - males from the low-quality treatment fertilised fewer eggs than males from the high-quality treatment (*t*_75_ = − 2.15, *p* = 0.035; block *p* = 0.237; Fig. 6).

### Effect of juvenile diet quality on male-induced harm

**Fig. 7:**
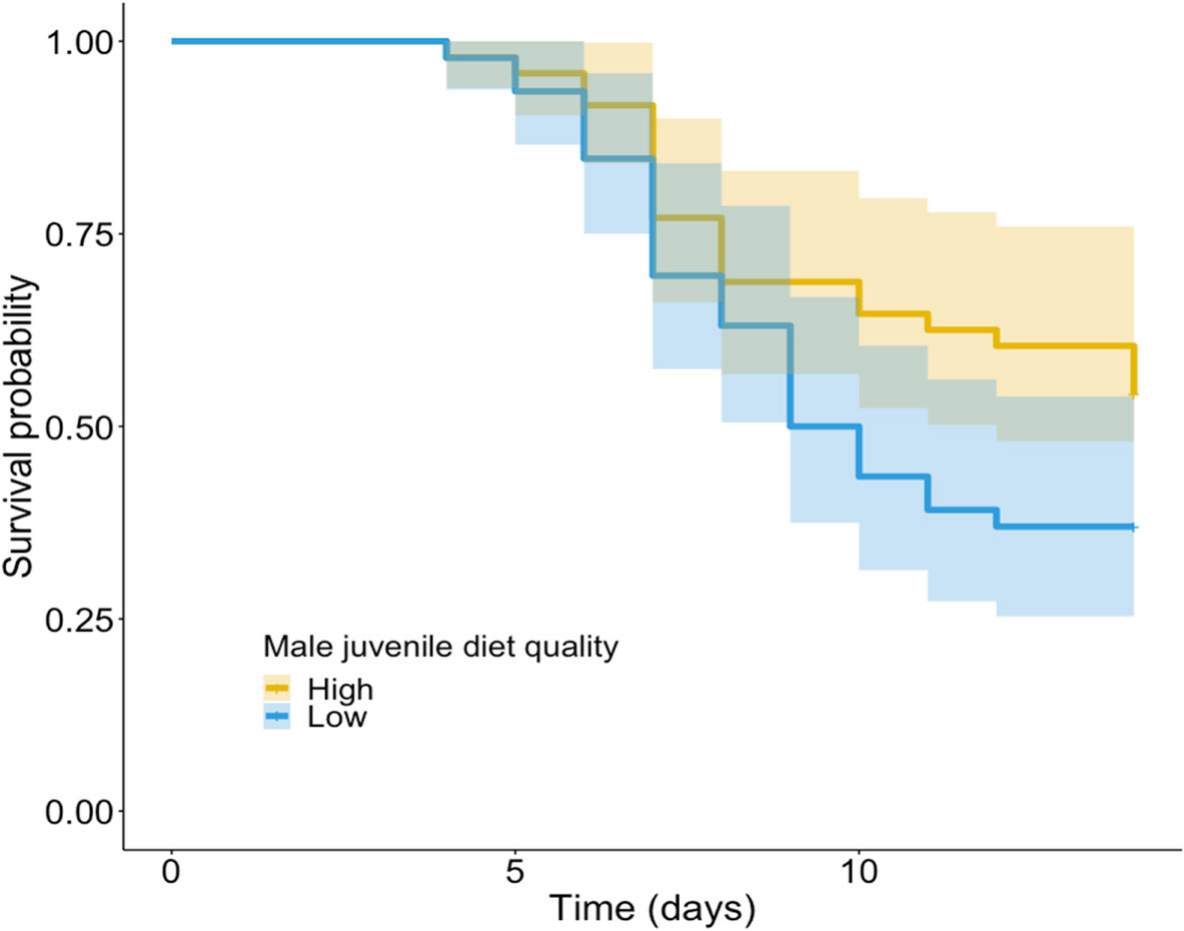
Effect of male diet quality during development on female lifespan. Kaplan-Meier survival plots for females maintained with males in the high- and low-quality treatments

**Fig. 8:**
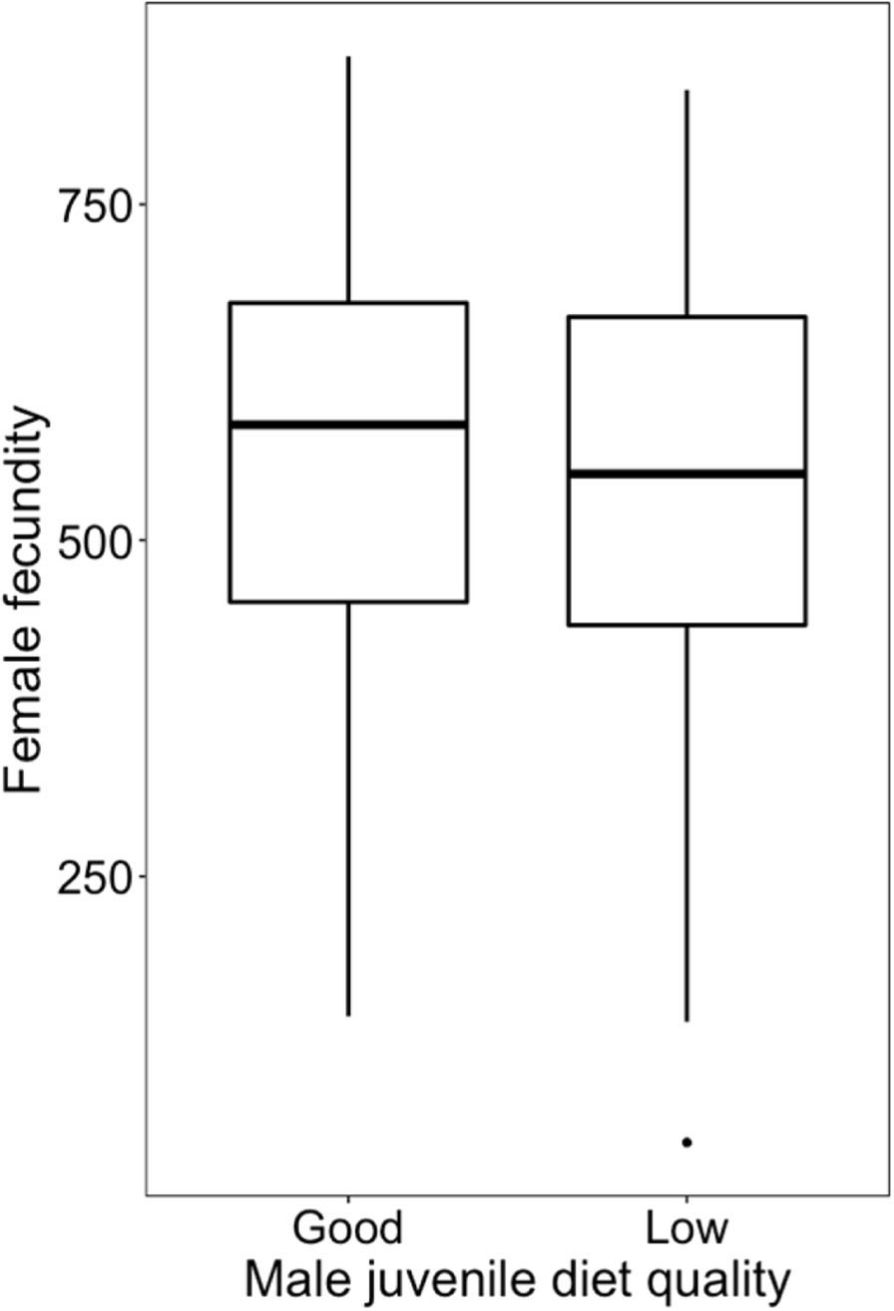
Effect of male diet quality during development on female reproductive output. Boxplots showing the distribution of the number of eggs laid (between the 1st and 6th days of the experiment) by females mated with males in the high- and low-quality treatments. The box encloses values between the first and third quartiles of the data (the inter-quartile range, IQR), while the horizontal bar within the box indicates the median. Whiskers extend from the box to the largest/smallest values that are within 1.5Χ the IQR of the box. Values outside that range are outliers and are indicated by circles

Contrary to expectations, females mated with males from the low-quality treatment exhibited lower survival than females mated with males from the high-quality treatment, but this trend was not significant (z = 1.724, *p* = 0.085; Fig. 7). There was no significant effect of male diet quality on female fecundity (t = 0.899, *p* = 0.370; Fig. 8).

### Effect of juvenile diet quality on female resistance to male-induced harm

**Fig. 9:**
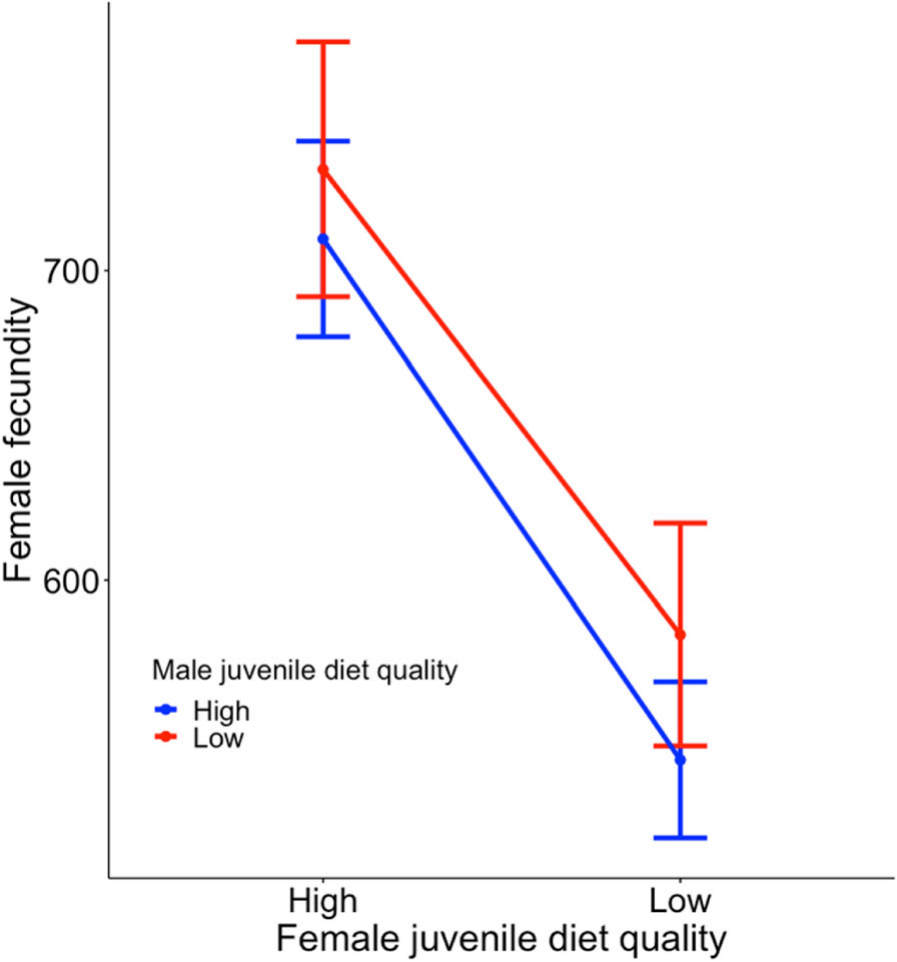
Effect of female diet quality during development on their reproductive output Mean number of eggs (=/− SE) laid by females (between the 1st and 6th days of the experiment) fed with high-quality food and low-quality food during development after 3 days of mating with males fed with high-quality food and low-quality food during development

Females treated with a high-quality diet laid a significantly high number of eggs (F_1,103_ = 11.834, *p* < 0.001, Fig. 9), but the male juvenile diet had no effect (F_1,103_ = 0.216, *p* = 0.643, Fig. 9). The interaction between the factors was non-significant (F_1,103_ = 0.069, *p* = 0.793; Fig. 9).

## Discussion

Despite the general agreement about condition dependence in the expression of sexually selected traits [44, 49, 50], our understanding of this mechanism in sexual conflict dynamics is currently limited. Here I subjected mites *S. berlesei* to different juvenile diet and found that the ability of males to allocate resources may affect the variation in traits involved in male-male competition and male competitiveness, consistent with previous reports [39, 51, 52]. However, my results do not support the hypothesis that food quality during development, via the individual’s condition, can modulate the intensity of sexual conflict.

### Sexual conflict in Sancassania berlesei

High mating frequency may have both benefits and costs for female fitness. Previous research on *S. berlesei* showed that females reared in a population with a strongly male-biased sex ratio exhibited decreased longevity and fecundity in comparison to kept under monogamy [48]. However, the strongly biased sex ratio (5:1) might have unnaturally increased the mating frequency and/or sexual harassment. In this study, females from the high-mating-rate group lived shorter lives than females from the LMF treatment (Fig. 1, Table 1) and laid fewer eggs due to decreased lifespan (Table 2), indicating that frequent mating is costly to females also under a 1:1 sex ratio. Moreover, the observed decrease in female lifespan was driven by the interaction between mating frequency and male morph (Fig. S1, Table S1). From the female perspective, mating with scrambler males entails different costs when the mating frequency differs, whereas such an effect was not observed in the case of mating with fighter males. Such conflict over mating is expected to be common in species like *S. berlesei*, characterised by condition-dependent reproductive tactics [53]. However, only few empirical studies paid attention to effect of mating with different male alternative phenotypes on female’s costs [54–58]. Recent studies showed that variation in population density, which is the key cue in morph determination in *S. berlesei*, may play an important role in mating costs experienced by females in species with alternative reproductive tactics [57]. It may suggest that full understanding of sexual conflict requires the consideration of interactions between females and both male phenotypes separately.

The costs of mating, overall, might be caused by male activity associated with copulation (e.g., traumatic insemination [59]). However, this scenario seems unlikely due to the biology of *S. berlesei*. As was discussed in Radwan & Rysińska (1999) male mating behaviour does not hurt or damage females. In contrast to the situation in a related species, *Rhizoglyphus robini*, costs associated with post-copulation guarding are reduced because such behaviour is not observed in *S. berlesei*. Moreover, time costs due to copulation are low since females are able to eat during copulations. The other cause of the observed mating costs might be post-copulatory male-induced harm. Such harm may be driven by accessory substances transferred with male ejaculate, such as in *D. melanogaster*, where male seminal fluids boost short-term female fecundity at the expense of their lifespan and lifetime reproductive success [60, 61]. Presented here results may suggest that similar process might work in the mite, as well. *S. berlesei* males, similar to *D. melanogaster*, transfer some fluid which helps displace sperm of a female’s previous mates [47, 62]. However, details on the composition of the ejaculate and the function of accessory substances in Acaridae mites are lacking. Further research in this area in a system other than insects may contribute to a better understanding of the adaptive nature of seminal fluids and their role in mating costs in females.

### Ecology of sexual conflict - effect of food quality

For decades, the role of ecology in sexual conflict research has largely been ignored. Recent studies have provided important information regarding multiple ways in which ecological factors can affect antagonistic coevolution and evolutionary outcomes of sexual conflict in water striders [27, 63]. It has also been shown that factors such as temperature [30] and physical environment [29] modulate the intensity of sexual conflict in *D. melanogaster*. Other potentially important environmental factors are food quality and availability. Experimental evolution under random and regular food availability revealed sex specific reduction in male lifespan which was compensated for increased reproductive success when food was provided randomly [36]. In contrast, female fitness did not change between evolutionary regimes, which suggests that sex-specific changes to male life history traits lead to reduced sexual conflict [36]. Rapkin et al. (2016) showed that the ratio of protein and carbohydrate intake by male decorated cricket (*Gryllodes sigillatus*) is important for regulation of the weight and gustatory appeal of nuptial gifts—traits that are involved in mediating sexual conflict in this species [64]. Nevertheless, our knowledge of the ecology of sexual conflict remains poor. In this study, I evaluated whether diet quality during development affects traits involved in sexual conflict and thus whether the intensity of sexual conflict is condition dependent in the mite *S. berlesei*.

Since adaptation to male-male competition may affect male-induced harm towards females, I first investigated the effect of food quality during development on male competitiveness and reproductive success. I found a significant effect of the juvenile male diet on adult sexual behaviour measured as time to start of mating and number of sexual interactions during a 60 min observation (Figs. 3 and 5). Since in *S. berlesei* the last male sperm precedence was observed and the proportion of eggs fertilised by focal males increased with the time the female took to re-mate with another partner [65], variation in measured sexual behavioural traits may influence the male reproductive success. Indeed, the results support the idea that diet quality during development affects the ability of males to father offspring with non-virgin females (Fig. 6).

These results are consistent with predictions that expression of costly sexual traits, including traits potentially linked to sexual competitiveness, decreases when the quality of the food resources is low [44, 66]. This prediction was also confirmed in insects when the food regime was manipulated during adulthood [51] as well as during larval rearing [39, 52]. Other explanations, however, cannot be excluded. Since the diet manipulation used in this research had a strong effect on male body size, it might in turn result in a decrease in sperm cell production. If sperm count determines sperm competition ability in *S. berlesei,* males fed a low-quality diet may fertilise fewer eggs than those fed the high-quality diet.

Contrary to the prediction that less competitive males should be less harmful to females, I found a non-significant negative effect of poor male diet on both female fecundity and lifespan (Figs. 7 and 8). The lack of effect of male diet quality during development on female fitness can be explained by the potential absence of ‘real’ low-quality genotypes in the low-quality treatment due to increased mortality. Alternatively, and perhaps more likely, the decreased number of sperm cells due to male body size would explain the lack of differences in female fecundity between treatments. In this context, it is possible that low-quality males are worse at providing enough sperm cells to fertilise all female eggs, which may diminish the positive effect of reduced male-induced harm in the low-quality treatment.

Sexually antagonistic coevolution can proceed as an evolutionary ‘arms race’ between the sexes [2], where the evolution of male persistence leads to the evolution of female resistance, which should generally not be limited to single traits. Theoretical research suggests that resistance should be affected by a range of traits related to female morphology, physiology and behaviour [1]. Condition dependence of such traits will select for females with increased resistance to male-induced harm and provide fuel for an ‘arms race’ between the sexes. The results of this experiment revealed that the diet of *S. berlesei* females during development affects fecundity, but the diet of males or the interaction between these two factors had no significant effect (Fig. 9). A recent study by Iglesias-Carrasco et al. (2018) showed a similar pattern. Females of *Callosobruchus maculatus* in good condition laid more eggs and lived longer; however, there was no interaction between the male and female condition states, suggesting that increased cost of mating with good condition males is not mitigated by the female condition [67]. In contrast, it is highly likely that the non-significant interaction observed in *S. berlesei* results from the lack of difference in male-induced harm between the low- and high-quality treatments.

## Conclusions

In conclusion, manipulation of juvenile food resources had no impact on neither male-induced harm nor female resistance in studied species, despite the effect on male competitive abilities. Nevertheless, similar studies on other species and ecological settings are needed to understand how environmental factors via individuals condition impact the evolution of male competition, female harm level and sexual conflict in natural populations, as well as how this phenomenon feeds back into population viability.

## Methods

### Study species

The mites came from a laboratory-adapted base population collected near Stirling in central Scotland in 1998 and have been maintained at large population (> 1000 adult individuals) since collection [68]. In the laboratory, mites were maintained at 22–23 °C and > 90% relative humidity and fed with dried yeast twice a week. Obtaining virgin individuals for the experiments was achieved by collecting quiescent (immobile) mites in the second or final juvenile stage (protonymph or tritonymphs, respectively) from the stock population and rearing them in isolation. Two male morphs occur in *S. berlesei*: aggressive heteromorphs (fighters) and nonaggressive homeomorphs (scramblers). Because male morph determination takes place at the early tritonymphal stage [69] and is based on population density cues [70] and/or body size and weight [71, 72], selecting a particular stage prior to isolation helped to control the proportion of fighters among adults. The mites were reared individually in glass tubes (Ø 8 mm) with bases made with plaster of Paris and charcoal and closed with cotton stoppers. All tubes were kept on Petri dishes (Ø 100 mm) that were covered with filter paper moistened once a day with tap water. The filter paper was replaced once a week. The same conditions were maintained throughout the experiments described below.

### Effect of mating frequency on female fitness

All females and males used in the following experiment were obtained by isolating quiescent protonymphs and tritonymphs, the last juvenile stage, from the stock population into individual vials. Between 12 and 24 h, after the final moulting, all females were randomly subdivided into two treatments with different mating frequencies. In the high and low mating frequency treatment (HMF, *n* = 59; LMF, n = 59, respectively), each female had access to a male for 20 or 4 h a day. Since copulations of non-virgin individuals were observed frequently in small populations [62] as well as in monogamous pairs, even after 2 days of interaction [48], I could assume that females that had access to males for longer period of time mated more often.

Each male was transferred from HMF treatment to LMF treatment for 4 h, daily; after that time males were transferred back to initial vial within HMF treatment. Using the same male in both treatments allowed me to show in a more effective way how the same individual affects female fitness depending on differences in mating frequency. To control the effect of male morph on female fitness, I used both fighters (eclosed from protonymphs; *n* = 29) and scrambler males (eclosed from tritonymphs; *n* = 30). Females were always paired with the same male throughout the experiment unless he died. Dead males were replaced with new ones of the same morph and age; mating with more than one male was recorded. To avoid adverse impacts of manipulation on female survival, females were transferred to new vials only twice - on the 6th and 11th days of the experiment.

Females were checked daily for survival for the entire duration of the experiment (18 days). Survival analyses were performed using a mixed-effect Cox model with male identity as a random factor and treatment, male morph, interaction between treatment and male morph as well as mating with more than one male as a fixed factors (function *coxme*, R package *coxme* [73]). The results from the mixed-effect Cox model revealed a significant interaction between mating frequency treatment and male morph. To investigate this interaction in detail, I conducted a separate mixed-effect Cox model for fighter and scrambler morphs.

As a measure of female fecundity, I used the number of eggs laid between the 6th and 11th days of the experiment, as a preliminary survey showed that the number of eggs laid during this period is highly correlated (r = 0.94, *n* = 41, *p* < 0.001) and represents 47% of stock female lifetime fecundity. Female fecundity was analysed using a generalised linear mixed model (GLMM) with a negative binomial error distribution (function *glmmadmb*, R package *glmmADMB* [74, 75]) to account for overdispersion. Male identity was introduced into the model as a random factor, and treatment, male morph, female longevity and the treatment * male morph interaction were fixed factors. Seven females (three, three and one in fighter HMF, scrambler HMF and scrambler LMF treatment, respectively) that died before the 6th day of the experiment were excluded from the fecundity analysis. The goodness-of-fit of the models was evaluated using the Akaike information criterion (AIC) [76] in both the mixed-effect Cox model and GLMM.

### Manipulation of juvenile diet quality

I started the following experiments by collecting the quiescent protonymphs from the stock population and housing them individually. Individuals were randomly subdivided into two diet treatments: low-quality diet and high-quality (control) diet. Mites from the high-quality treatment were fed with dried yeast ad libitum; the low-quality treatment contained filter paper, which is considered to be a reduced-quality food [77] and one grain of dried yeast (dimensions ca. 0.25 × 0.5 × 0.25 mm). Between 12 and 24 h before adults emerged, all individuals from both treatments were fed with the same amount of dry yeast (3–4 grains) to avoid the effect of starvation on the measured traits. Virgin individuals 12 to 24 h after the final moulting were used for experiments to avoid possible differences due to age.

To investigate whether diet treatment influenced the condition of an individual, the body length (excluding mouthparts) of virgin adult males was measured from digital images using ImageJ software, version 1.52a (Rasband, W.S., ImageJ, U. S. National Institutes of Health, Bethesda, Maryland, USA, https://imagej.nih.gov/ij, 1997–2018). The effect of treatment on body length was analysed with the Mann-Whitney U test. Since the low-quality diet used in this part suppressed the expression of the fighter morph (90% of obtained males were scramblers), only scrambler males were used in all subsequent experiments.

### Effect of juvenile diet quality on male sexual behaviour and competitiveness

To assess how diet influences male competitiveness and fertilisation success, I used a male sterilisation-based method [62, 78]. Virgin males from the stock population were irradiated with 120 Gy gamma rays (^137^Cs source giving approximately 3.55 Gy/min; Gammacell® 1000 Elite, BestTheratronics Ltd., Canada). After that dose, irradiated male sperm can fertilise eggs, but the eggs exhibit 100% embryonic mortality; thus, the eggs remain unhatched. Virgin females from the stock population (*n* = 78) were paired with males in the following order: irradiated male, focal male, irradiated male. This triple-mating experiment allowed to test male competitiveness in conditions similar to that in a large and promiscuous population of *S. berlesei*. In such populations sperm cells may regularly compete with sperm of both previous and next female’s mating partner.

The fertilisation success of the focal males was measured as the proportion of hatched eggs. Each male was kept with a female for 60 min, ensuring the occurrence at least one copulation event (personal observation). During the 60 min of observation, I recorded the number of copulation attempts, the time to start of the first copulation and the time spent on copulation by a focal male. For the next 3 days, the females laid eggs, and after 5–7 days, the proportion of eggs that hatched was determined. Due to logistical constraints, the experiment was performed in two blocks with 18–20 replicates of each experimental treatment in each block.

The proportion of eggs sired by focal vs irradiated males was analysed using a generalised linear model (GLM) with quasibinomial errors to account for overdispersion [79]. The number of larvae and unhatched eggs were treated as two-vector response variables [79]. Three observations were removed from the analysis because the females did not lay any eggs. Statistical analyses of male sexual behaviour were performed using the GLM with Poisson error for the number of copulations and linear model for the time to start of mating and mating duration. The time to start of mating was log-transformed to improve the normal distribution of the residuals. Distribution of residuals were checked with diagnostic plots and Shapiro–Wilk test. Due to missing values, one, five and one observations were removed from the analysis of the number of copulations, time to start of mating and mating duration, respectively. All models included treatment (low- vs. high-quality diet) and block as fixed factors.

### Effect of juvenile diet quality on male-induced harm

To examine how the male condition affects female reproductive output and lifespan, I paired virgin females from the stock population with a virgin male from the high-quality (*n* = 48) or low-quality (*n* = 45) food treatment in a 0.8-cm-diameter glass cell. On the second and sixth days of the experiment, I replaced the males with new ones from the same treatment. Allowing the female to copulate with more than one male decreased the possible effect of the male on fecundity due to occasionally observed male sterility.

The experiment was maintained for a 14-day period during which females were checked daily for survival. Survival analyses were performed using the Kaplan-Meier survival estimate and Cox proportional hazards model by comparing survival curves (function *survfit coxph*, R package *survival* [80, 81]). Due to the logistical constraint and the time-consuming process of egg counting, the effect of diet quality treatment on female reproductive output was examined using the number of eggs laid between the 1st and 6th days of the experiment. This continuous 6-day recording period is considered to be representative (44%) of female lifetime fecundity due to high positive correlation (r = 0.887, *n* = 41, *p* < 0.001) with lifetime fecundity in the stock population of this species (results from preliminary experiments). Female fecundity data were tested for normality using the Shapiro–Wilk test and for equality of variance using Bartlett’s test separately for each treatment level. Given that the normality and equality of variance assumptions were met, the mean number of eggs laid by females was compared between treatments with a t-test.

### Effect of juvenile diet quality on female resistance to male-induced harm

To determine the effects of treatment on female resistance to male-induced harm, I used a full-factorial experimental design. Females from the high- and the low-quality food treatments were mated with males from both experimental procedures for 3 days. Then, the males were removed, and the females were allowed to lay eggs for the next 3 days. Female fecundity was estimated based on the total number of eggs laid by females over 6-day period. The differences in the total number of eggs laid by females were analysed by two-way ANOVA, as the normality and equality of variance assumptions were met, and with type III sums of squares due to differences in the number of replicates (27–29 females per experimental group).

All statistical analyses were performed in R version 3.4.1.

## Supplementary information

**Additional file 1: Figure S1.** Effect of mating frequency and male morph on female lifespan. Kaplan-Meier survival plots for females maintained in high mating frequency or low mating frequency treatment and mated with scrambler or fighter male morph. **Table S1.** Results of a mixed-effect Cox model of female survival probability as a function of mating with a scrambler or fighter in high mating frequency and low mating frequency groups, with experimental treatment and number of mated males as fixed effects and male identity as a random factor.

## Data Availability

The datasets used and/or analysed during the current study are available from the corresponding author on reasonable request.

## Abbreviations

GLM: Generalized linear model
GLMM: Generalised linear mixed model
HMF: High mating frequency
LMF: Low mating frequency
