# Supplementary material for: Juvenile diet quality and intensity of sexual conflict in the mite Sancassania berlesei
Source: BMC Evol Biol. 2020 Mar 12;20:35. doi: 10.1186/s12862-020-1599-5 (PMC7069193; doi:10.1186/s12862-020-1599-5)
Supplement: Supplementary file 1 — Additional file 1: Figure S1. Effect of mating frequency and male morph on female lifespan. Kaplan-Meier survival plots for females maintained in high mating frequency or low mating frequency treatment and mated with scrambler or fighter male morph. Table S1. Results of a mixed-effect Cox model of female survival probability as a function of mating with a scrambler or fighter in high mating frequency and low mating frequency groups, with experimental treatment and number of mated males as fixed effects and male identity as a random factor. [file 12862_2020_1599_MOESM1_ESM.docx]

**
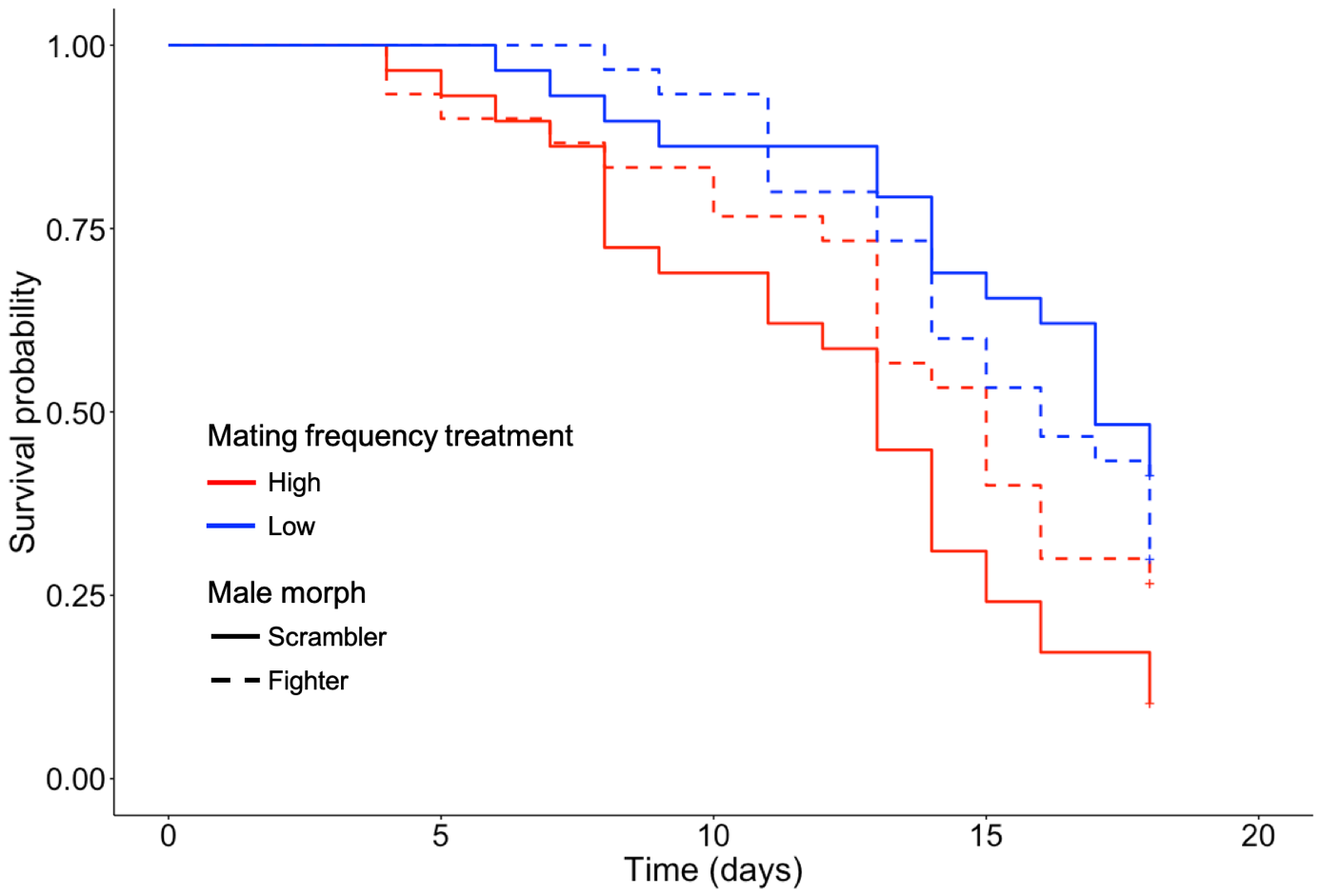
**

**Fig. S1.** **Effect of mating frequency and male morph on female lifespan.** Kaplan-Meier survival plots for females maintained in high mating frequency or low mating frequency treatment and mated with scrambler or fighter male morph.

| *Female mated with:* | *Scrambler* | | | | | |  | | *Fighter* | | | | | | |
| --- | --- | --- | --- | --- | --- | --- | --- | --- | --- | --- | --- | --- | --- | --- | --- |
|  | *coef.* | *s.e* | | *z* | *p* | | | |  | *coe.f* | *s.e* | | *z* | *p* | |
| ***Fixed effects*** |  |  | |  |  | | | |  |  |  | |  |  | |
| Treatment | -1.30 | 0.35 | | -3.74 | **<0.001** | | | |  | -0.28 | 0.31 | | -0.91 | 0.36 | |
| Mating with more  than one male | -0.99 | 0.47 | | -2.11 | **0.035** | | | |  | 0.48 | 0.39 | | 1.21 | 0.22 | |
| ***Random effect*** | *variance* | | *std. dev.* | | |  | | | *variance* | | | *std. dev.* | | | |
| Male identity | 0.508 | | 0.713 | | | | |  |  | 0.153 | | 0.39 | | |  |
| Abbreviations: *coef.*, coefficient; *s.e*, standard error; *std. dev.,* standard deviation; *p* < 0.05 highlighted in bold. | | | | | | | | | | | | | | | |

**Table S1.** Results of a mixed-effect Cox model of female survival probability as a function of mating with a scrambler or fighter in high mating frequency and low mating frequency groups, with experimental treatment and number of mated males as fixed effects and male identity as a random factor..
